# Anæsthetisation for Operations on the Central Nervous System

**Published:** 1908-06-13

**Authors:** 


### Anæsthetics.

### ANÆSTHETISATION FOR OPERATIONS ON THE CENTRAL NERVOUS SYSTEM.

There are few branches of surgery in which more astounding progress has been made in recent years than in the operative treatment of various central nervous lesions. The localisation of cerebral and spinal tumours, abscesses, and cysts; the various devices for dealing with intractable neuralgias of many kinds, from division of nerve trunks or of posterior nerve roots to the removal of the Gasserian ganglion; the modern technique for dealing with lateral sinus thrombosis, tuberculosis of the vertebræ, depressed fractures of the skull, and many other conditions; all these have added enormously to the number of operations performed on the central nervous system and its bony surroundings.

The problems that arise in inducing and maintaining anæsthesia in these cases are therefore no longer the monopoly of one or two consulting anæsthetists in large towns. Any one whose practice includes anæsthetic work for fellow practitioners may be faced with a case of one of the kinds just mentioned, especially in a place large enough to support a general, or even a cottage, hospital. There are certain features in which, broadly speaking, the requirements of surgery and of the patient differ in these central nervous cases from the ordinary run of anæsthetic work. For one thing the very centres from which respiration and cardiac function are controlled may be already injuriously affected by the lesion for which the operation is to be performed. The intracranial pressure of a cerebral tumour may, as is well known, produce a very marked slowing of the pulse, and it may also cause a condition of unstable equilibrium in the respiratory centre, which a very slight addition of anæsthetic toxæmia may convert into absolute disorganisation. There are, in fact, many operations on the skull and spine in which large alterations of tension are quickly produced in the cerebro-spinal fluid.

Fortunately the depth of anæsthesia required for most of these operations is comparatively very slight. If it were necessary to induce anæsthesia to the same degree as for many abdominal operations the danger of the proceeding would be almost altogether prohibitive. For one thing, ether is practically out of court as an anæsthetic for nervous cases, at least by any of the "closed" methods of administration. Spinal anæsthesia by lumbar injection is also evidently useless for the majority of cases. At one time it was frequently recommended to give sufficient doses of morphia or opium to reduce the patient to semisomnolence before the administration is begun, but this is a method which has distinct dangers of its own, and is not now very often practised. The anæsthetic *par excellence* for an operation on the central nervous system is chloroform, partly because an extremely light anæsthesia can be more easily maintained than by other drugs, partly because there is no limitation of air entry with it, and partly because there is no engorgement of the blood-vessels whereby bleeding and obscuration of the surgeon's field of view may occur. During induction, which may be procured by the C.E. mixture if preferred, the patient is best in the ordinary supine position with the head turned to one side. The usual precautions to ensure that he has a free airway must, of course, not be neglected. As a rule, one must be prepared for a cessation of the administration for a variable time while the patient's position is adjusted to suit the requirements of the surgeon, and therefore it is often necessary, before giving permission for this, to carry the anæsthesia to a depth rather greater than that at which it will be subsequently maintained; as far, for instance, as for an ordinary major operation on a limb, but not as for one on the abdomen.

When the various sandbags, arm-rests, head-rests, and so on are arranged, the anæsthetist will often find the patient's face and upper air passages in an extremely awkward attitude for the inhalation of the anæsthetic. This he must endeavour to rectify as far as possible before any more of the drug is exhibited, and it is sometimes necessary in spinal cases to let down the head of the operation table altogether and support the prone patient's head on a towel spread on one's lap. In many cerebral cases where the field of operation is far forward the anæsthetist must be prepared to have his hands, his apparatus, and his patient's face entirely buried in sterilised towels, and the consequent restriction of air entry and inability to watch the patient's eyes and lips are often formidable difficulties. One of the great advantages of the Vernon-Harcourt inhaler for these patients is the long tube, which can be attached to the face-piece, and permits of free access of air to the patient when the face is completely enveloped in towels.

Anæsthesia must be maintained at a distinctly low pitch. There is the gravest danger in anything more than this during an operation on the brain or spinal cord. The ideal to aim at is just not to allow purposive movement and phonation, though a mild degree of either is no great detriment, and is a departure on the side of safety. The patient is extraordinarily susceptible to very slight changes in the amount of chloroform exhibited, and in the quantity and quality of the inspired air. For these reasons there is no doubt that the Vernon-Harcourt inhaler is of very great value in dealing with operations on the central nervous system. The great drawback to its use is the very prolonged induction period, but this can be got over by the use of an ordinary domette mask and drop bottle. When once the patient is ready for the surgeon the face-piece of the inhaler is adjusted, and the anæsthetist can then view with unconcern any alteration in the patient's position and any amount of swaddling in dressings or towels.

This face-piece is of rubber, and is similar to that of a Clover's inhaler; it is essential that it be adjusted to fit with absolute accuracy. From it an indiarubber tube guarded by an outer coil of wire leads to the inhaler itself, which, for convenience, is best mounted on a stout, broad-based pillar instead of being held in the hand, as in the earliest patterns. The principles of the apparatus we need not enter into, but it is important to keep up the temperature of the chloroform chamber by immersing it in a vessel of warm water. Otherwise it becomes chilled by evaporation, and the indicator is no longer accurate. The quantity of chloroform vapour to be delivered varies in individual cases, but is generally between 0.6 and 1.4 per cent. The rapidity with which the patient responds to very slight changes in this percentage is remarkable, and shows the danger of drop-bottle methods in these particular cases.

In all operations on the central nervous system there is especial danger of asphyxial complications, the onset of which is speedily made evident in the colour of the blood as it oozes out of the cut tissues. To meet this condition promptly is of great importance, and is best attained by giving oxygen with the chloroform. This can be done by having a cylinder of the gas in communication with a T-piece and stop-cock on the inhaler close to the connection with the tube leading to the face-piece. Another point which must be carefully borne in mind, especially in cases where the anæsthetist cannot see the patient's face, is not to mistake asphyxial for purposive movements. The former are commonly slight backward jerky movements of the head often associated with laryngeal stridor, and call for the immediate exhibition of oxygen and the temporary suspension of the anæsthetic.

When an inhaler of this pattern is not at hand the best plan is to use an ordinary Skinner's mask and drop bottle. There is need for great vigilance throughout, and but very small quantities of the drug should be dropped on the mask at a time. The surgeon should not be allowed to interfere in any way with a clear airway, and the corneal reflex should on no account be allowed to disappear. If there is no especial objection to the secretion of a certain amount of mucus, as there would be in the subject of nasal obstruction, for example, ether may be given by the open method. One disadvantage of this in operations about the head is the inconvenience to the surgeon of working in an atmosphere heavily charged with this vapour. The anæsthetist is to a great extent acclimatised to this drawback, but cranial surgery is often hard and exhausting work, and demands a clear head. Whatever be the method selected, the key to success is to maintain the lightest of anæsthesia, and to prevent the least degree of asphyxiation. In these two respects does the anæsthetisation of patients for operations on the central nervous system differ most from that for other procedures. Before leaving his patient, and also during the operation, the anæsthetist must take particular care that warmth is secured by blankets, hot bottles, and so forth, to avoid the considerable shock which often follows these severe operative measures.

